# Why so Few, Still? Challenges to Attracting, Advancing, and Keeping Women Faculty of Color in Academia

**DOI:** 10.3389/fsoc.2021.792198

**Published:** 2022-01-18

**Authors:** Jean E. Fox Tree, Jyotsna Vaid

**Affiliations:** ^1^ Psychology Department, University of California Santa Cruz, Santa Cruz, CA, United States; ^2^ Psychological and Brain Sciences, Texas A&M University, College Station, TX, United States

**Keywords:** women of color, scholars of color, academia, barriers, hiring, retention

## Abstract

From its earliest beginnings, the university was not designed for women, and certainly not for women of color. Women of color in the United States are disproportionately under-represented in academia and are conspicuous by their absence across disciplines at senior ranks, particularly at research-intensive universities. This absence has an epistemic impact and affects future generations of scholars who do not see themselves represented in the academy. What are the barriers to attracting, advancing, and retaining women faculty of color in academia? To address this question we review empirical studies that document disparities in the assessment of research, teaching, and service in academia that have distinct implications for the hiring, promotion, and professional visibility of women of color. We argue that meaningful change in the representation, equity, and prestige of women faculty of color will require validating their experiences, supporting and valuing their research, creating opportunities for their professional recognition and advancement, and implementing corrective action for unjust assessment practices.

## Introduction

The university as an institution was founded by and largely for men and, in particular, for White men (Thelin et al., 2021). Particularly in elite universities in Europe and the United States, women (White or other), and racialized groups (of any gender) were not allowed to pursue higher education or be employed as faculty until fairly recently (Lewis, 2019; Vaid and Geraci, 2016). Moreover, in the United States the intertwinement of the history of the university with the history of enslavement (Wilder, 2013) has contributed to a further entrenchment of beliefs (even in the academy) that perpetuate notions of white supremacy. Relatedly, the social construction of White (male)-as-default by professional societies and academic journals in the behavioral and social sciences has shaped what is seen as mainstream (and by implication, meritorious) research (Buchanan et al., 2020). Thus, even decades after women and racialized groups have entered the academic workforce there is no parity in their representation or salary and their professional advancement has been uneven and slow (Valian, 1998). Women have been regarded as “outsiders in the sacred grove” (Aisenberg and Arrington, 1988) and women of color have had to repeatedly prove their right to belong in the academy (Williams, 2014), being “presumed incompetent” (Gutierrez y Muhs et al., 2012).

As a recent analysis of the behavioral and social sciences research workforce based on 2013 NSF data showed, although there is gender balance in some disciplines (e.g., psychology and sociology), others remain male dominated (economics and political science). Yet even in psychology, a discipline that has had gender parity for several decades (American Psychological Association, 2006), the percentage of women in full professor positions remains lower than that of men (American Psychological Association, 2014). Moreover, relative to the other social sciences, psychology has the highest proportion of White faculty. In 2013, the overall United States population of non-Hispanic Whites was 67% but the percent of non-Hispanic White faculty in psychology was 85%; the percent of Blacks and Hispanics in the overall United States population was 12 and 14%, respectively, and the corresponding figures among psychology faculty were 5% in each case (Hur et al., 2017). Indeed, relative to biomedical and engineering disciplines (which have less gender diversity), behavioral and social science researchers have less racial and ethnic diversity (Hur et al., 2017).

From getting in to a PhD program (Maton et al., 2006), getting graduate research support (Sheppard et al., 2001; Bartolone et al., 2014), finishing a PhD program (Maton et al., 2011), securing an academic job (Pico et al., 2020) or a federal research grant (Ginther et al., 2011), and getting promoted through the ranks (e.g., Dutt et al., 2016), people who have historically been excluded from academia have faced many obstacles. Decades of efforts have not made a noticeable difference in terms of representation or recognition (e.g., Stewart and Valian, 2018; Bennett et al., 2020; Vaid and Fox Tree, 2020).

Women faculty of color in academia are particularly conspicuous by their absence. In every STEM discipline, and particularly in the behavioral and social sciences academic workforce, women of color (particularly Black and Latina women) are disproportionately under-represented relative to their percentage in the overall population. In the United States, data from Fall 2005 showed that—across all ranks—the representation of women among full-time tenured or tenure track faculty (467,325) included 2.33% Black (10,879), 2.34% Asian (10, 944), 1.20% Latina (5,606), and 28.9% white (135,158) (Chronicle Almanac 2007–2008, 2008, p. 25). Viewed by rank, a 2015 report by the National Science Foundation found that Black/African American, Latina, and Native women in the United States collectively accounted for fewer than 1% of full professors, fewer than 2.5% of associate professors, and fewer than 3% of assistant professors (National Science Foundation, 2015). Relatedly, in the United Kingdom, of a total of 21,000 professors in 2020 there were only 25 female black professors (Agunsoye, 2020).

Why are there so few women faculty of color in positions of power and prestige in academia? Is it because they do not meet the standards expected of those in such positions? We do not think this is the case. We argue, instead, that the persistent absence of women of color in academia reflects systemic inequities reproduced and reinforced by the culture of academia and its discourse of meritocracy. As Carter-Sowell et al. (2019, p. 306) point out, the notion of meritocracy “masks ways in which certain groups have benefited and others have been excluded from access to resources and networks that lead to professional advancement.” We believe that explicit attention to structural barriers in academia will be helpful in providing a context to understand the challenges faced by women and by scholars of color in their quest to achieve equity in academia. As United States-based senior women faculty of color in the social sciences who are full professors we are members of that rarified club of 1 percenters. The status of women of color in academia, thus, has personal resonance for us. We hope it will also resonate with other women faculty of color whose voices and perspectives are all too often missing or given cursory attention in scholarship on the status and/or lived experiences of women in academia.

Compounding the absence of women of color in academia is an absence of scholarship that takes an intersectional lens on gender in academia (but see Corneille et al., 2019). Many United States-based studies on gender disparities among faculty in academia (in pay, recognition, productivity, impact, service loads, etc.) do not differentiate among different groups of women (e.g., Hur et al., 2017). Because most women in academia in the United States are White women, this means the studies that do not disaggregate by race/ethnicity are generally reporting on the experiences of White women. Similarly, studies of racial disparities in academia tend to focus on minoritized groups with little discussion of gendered experiences of these groups (e.g., Dimmick and Callahan, 2021). The practice of discussing gender without consideration of how gender intersects with racialized gender identities, or of discussing ethnicity or race without consideration of its gendered aspect, has contributed to the invisibility of women of color in academia as a subject of inquiry. This invisibility is reinforced by the way that research questions on women’s representation, equity, and prestige in academia have been framed and datasets coded. Thus, a crucial first step in addressing the persistent disparity in representation and prominence of women of color in academia is to acknowledge the dearth of intersectionality-oriented datasets and to push for more such data to be collected.

Because we cannot wait for the datasets to be reconfigured, we will consider existing scholarship on challenges faced by women in academia as a whole and those faced by scholars of color as a whole, recognizing that the challenges for women of color need not simply represent additive effects of being a woman plus being a person of color. Instead, the factors are likely to interact in unique ways. A fuller picture will come from intersectionality-informed quantitative data to supplement a growing number of first person accounts by women of color in the academy (e.g., Drame et al., 2012; Rollock, 2019; Buchanan, 2020; Chin, 2020; Comas-Dias, 2020; Niemann et al., 2020). These accounts bring up recurrent themes, including an unwelcoming institutional climate contributing to a sense of not belonging, being perceived as hypervisible and invisible, being asked repeatedly to prove one’s legitimacy as scholars or teachers, and being overworked and underpaid (see Carter-Sowell et al., 2019, for further discussion).

Although not usually configured intersectionally, there has been a veritable explosion of research on gender and/or ethnicity-related biases that may arise in faculty hiring, promotion and retention. A large, evidence-based literature has accumulated across an array of academic disciplines. In deciding which sources to include in this review we focused on recent studies and studies involving large-scale datasets. Where available, we have sought to highlight studies on academic psychology but have not restricted ourselves to that discipline.

Our review is organized as follows. We first discuss the challenges that women of color face in the hiring and promotion process, including how research, teaching and service are assessed. We believe that seeking equity in evaluating faculty can aid retention. Scholars feel more valued when their research work is recognized by the broader community. By the same token, they feel less valued and are more likely to leave academia when they feel their research is not recognized. Settles et al. (2021) refer to this devaluation of scholarship as *epistemic exclusion* and find that it is a predictor of turnover intentions among women and faculty of color. Relatedly, a sense of not belonging fostered by a chilly climate and not being in the information loop characterized faculty of color (men and women) in a climate survey conducted at a large research-intensive university (Zimmerman et al., 2016). The same study also found that women experienced more ostracism in the academic workplace than men faculty, and that this was irrespective of the percent of women in the department (Zimmerman et al., 2016). After reviewing challenges to hiring and promotion, we discuss possible interventions, bearing in mind that any interventions proposed need to be intersectionality-minded as well (see Liu et al., 2019, for further discussion of this point).

## Challenges to Hiring and Promotion

Challenges to hiring women and people of color come from biases that arise in research assessment, teaching assessment, and service assessment. These same biases come into play with promotion files. Understanding these biases is important because even small biases can lead to large differences. In a computer simulation where women were given a 1% downgrade to their performance evaluations, and where employees were successfully removed from the model until all the employees were new, the resultant organization was 65% male in the highest positions (Martell et al., 1996).

Please see Table 1 for a summary of the articles reviewed that tested disparities in research, teaching, and service.

**TABLE 1:** Summary of articles reviewed that tested disparities in research, teaching, and service.

	**Women Only**	**Scholars of Color Only**	**Both**
Research Impact	—	—	Hofstra et al. (2020)
Publication Process	Budden et al. (2008); Fox and Paine (2019)	—	—
Citation Rates	Caplar et al. (2017); DeJesus et al. (2021); Dion et al. (2018); Fox and Paine (2019); King et al. (2017); Lerchenmueller et al. (2019); Thelwall (2020)	Chakravarty et al. (2018)	—
Professional Recognition	Bendels et al. (2018); Ford et al. (2018); Nittrouer et al. (2018); Orchowski et al. (2021); Pico et al. (2020); Quadlin (2018); Treviño et al. (2018); Vaid and Geraci (2016); West et al. (2013)	Chakravarty et al. (2018); Roberts et al. (2020)	Ford et al. (2019)
Funding	Titone et al. (2018)	Erosheva et al. (2020); Ginther et al. (2011); Hoppe et al. (2019)	—
Recommendation Letters	Dutt et al. (2016); Madera et al. (2009); Madera et al. (2019); Steinpreis et al. (1999); Trix and Psenka (2003)	—	—
Training Institution	Clauset et al. (2015)	—	—
Teaching	Boring et al. (2016); El-Alayli et al. (2018); MacNell et al. (2015); Martin (2016); Mengel et al. (2019)	—	Chávez and Mitchell (2020); Pittman (2010)
Service	Guarino and Borden (2017); Misra et al. (2011)	—	Social Sciences Feminist Network Research Interest Group, (2017)

### Research Assessment

One of the biggest challenges in increasing representation of women of color in the professoriate is in evaluating research quality. Bias affects many aspects of research quality assessment. We begin this section by describing the contributions of women and scholars of color to science, and then describing the biases that affect evaluation of these contributions.

In what is probably the most comprehensive assessment of scientific innovation and the uptake of scientific ideas, Hofstra et al. (2020) evaluated the impact of all science dissertations produced in the United States from 1977 to 2015—over a million dissertations. The researchers used natural language processing techniques to identify topics in the dissertations, and then determined when those topics were first connected in a dissertation. They then looked at the uptake of those topic connections in future work. Women and people of color created more novel linkages. But while it was true that the more novel and impactful a PhD thesis, the more likely a scholar would have a research career, the work of women and people or color was not taken up to the same degree as work by men or White people. Women had about 5% lower odds of becoming faculty, and underrepresented minorities had 25% lower odds. Further, the more impactful the work, the greater the divide between women and men and underrepresented minorities and White scholars. Hofstra et al. (2020) dubbed this the *diversity-innovation paradox*. In essence, women and people of color were more likely to create novel connections in research work, but they were less likely to be rewarded for their innovation with research careers.

#### Disparity in the Publication Process

Some of the lack of recognition may stem from disparities in the publication process, resulting in the inability to get papers published or a delay in publication because of multiple rounds of revisions required. An assessment of over 23,000 manuscripts submitted to six ecology and evolution journals from 2010 to 2015 revealed gender parity in the articles submitted, but papers with women as first authors got lower peer review scores and were less likely to be published (Fox and Paine, 2019). While the authors made no claims about the causes of their observations, one possibility is bias in the review process. After a science journal started using double-blind peer-review, more papers with women first authors were published (Budden et al., 2008). Bias in the publication process may also arise if journal editors or reviewers make judgments about the fit (or lack of fit) of the submitted work with the journal’s intended scope or audience. Work that addresses groups that are not White or that is produced in a country that is less represented in academic literature may be considered not to be mainstream research and not suitable for mainstream outlets which typically have greater visibility than specialized outlets. As the primary metric of productivity and research prominence, the importance of publications cannot be overstated.

#### Disparity in Citation Rates

Even when papers are published, there is a lack of recognition of the contributions made by women and people of color. This is evident in lower citation rates of published reports across several disciplines. In political science, researchers observed undercitation of women scholars, although there was less of a gender gap the more women there were in the subfield (Dion et al., 2018). Undercitation of women’s papers was also observed in evolution and ecology journals (Fox and Paine, 2019) and astronomy journals (Caplar et al., 2017). Women also self-cite less than men; from 1991 to 2011, of 1.5 million JSTOR articles, men cited themselves 1.7 times the rate of women, with men’s higher self-citation rate persistent over time (King et al., 2017). While in the majority of papers there are no self-citations, the differential rate of self-citations can still lead to higher citation impact indices for men (King et al., 2017). Differences in citation rate also arise from differences in collaboration patterns, with multi-authored papers garnering more citations. The picture for people of color is similar. Researchers found that non-White scholars were underrepresented as authors in communication journals, and were also cited less often (Chakravarty et al., 2018). In contrast to these findings, an examination of six million papers produced between 1996 and 2018 showed that female first-authored papers were generally more cited than male first-authored papers, although citation rates were more even in the United States than in other English-speaking countries (Thelwall, 2020). At the same time, Thelwall (2020) proposed that female first-authored research was cited more because it had more societal implications. Thelwall (2020) also pointed out that evidence that female first-authored papers were cited more made the fact that women do not have parity in academia even more glaring.

Blind review would not necessarily remove citation rate discrepancies. Male lead authors used positive words like *novel*, *robust*, *excellent*, and *remarkable* in the titles and abstracts of their clinical research articles more than women lead authors (Lerchenmueller et al., 2019). Male lead authors also used more generic language than women lead authors (DeJesus et al., 2021). Generic language is overarching statements about groups, such as “Whites and Blacks disagree about how well Whites understand racial experiences” as opposed to focusing on how particular participants in a study behaved (DeJesus et al., 2019, p. 18,370). Papers using positive words and generic language were cited more often (DeJesus et al., 2021; Lerchenmueller et al., 2019). So, the discrepancy in citations may come about in part from the way men and women write about their work.

Citation counts matter because they introduce readers to the authors’ engagement with other authors’ thinking. As such, they are an important vehicle for bringing diversity of perspectives into a published work. Thus, it is not surprising that activist collectives such as Cite Black Women (see Smith et al., 2021) have discussed the importance and the politics of citation, both in terms of who cites whom and who tends to get erased by not being cited.

Citation counts are used as a proxy for impact and having lower citation counts adversely impacts the promotion of scholars. While it is important to increase citation to the work of women scholars of color, it is also important to note that citation counts may be lowest exactly for those doing cutting edge or non-mainstream work that has fewer researchers: Citation counts will be lower because there are fewer other scholars available to cite them.

#### Disparity in Professional Recognition

Tied to citation differences is author order and other markers of professional recognition. In a study of over 8 million research articles, researchers found that women were underrepresented in first and last author positions, as well as in single-authored papers (West et al., 2013). This assessment included natural and social sciences as well as humanities articles. Researchers who investigated almost 300,000 science articles found that women were more likely than men to be in non-prime author order on a multi-author paper (Bendels et al., 2018). Men were overrepresented in the prestigious last-author position, and particularly in the highest-impact journals (Bendels et al., 2018). In another study, women were found to be underrepresented as first authors in the thirteen top geoscience journals (Pico et al., 2020). Whatever the reasons might be that women do not seek first (or last) authorship to the same extent as men (e.g., perhaps they are more inclined to showcase student co-authors), these choices affect their professional recognition.

Beyond authorship practices, women and historically marginalized scholars suffer from a lack of professional visibility in other ways. One example is not being invited to give talks. An investigation of talks at a geophysical conference from 2014 to 2016 revealed that Black/African American, Latina/o/x, and Native American scholars were invited to give talks less often than White and Asian American scholars, and the situation was worst for underrepresented women (Ford et al., 2019). When men were the conveners of sessions, with control over who got a talk as opposed to a poster, they were less likely to give women talks (Ford et al., 2018). In another study, researchers evaluated how often over 3,000 speakers gave colloquium talks at one of the top 50 academic institutions in the United States in three social science fields, one natural science field, one engineering field, and one humanities field: men gave more talks than women, but if the colloquium host was a woman, women were more likely to give talks (Nittrouer et al., 2018).

Other examples of lack of professional visibility have also been noted. In cognitive psychology, a field that has had gender parity in doctorates for over 40 years, one would expect there to be gender parity in indicators of status and prestige. Yet, an analysis of various indicators as of 2015 showed that male cognitive psychologists were over-represented in professional society governance, as editors-in-chief of the top 60 journals in the field, and as recipients of prestigious awards (Vaid and Geraci, 2016). Across the field of psychology, a recent analysis found that women received less than a third of awards given out by the American Psychological Association across ten award categories over a 63 year period from 1956–2019 (Orchowski et al., 2021). Another study found that the majority of named awards given by the four leading professional societies in education are named after white men (Bazner et al., 2021). Among management professors, women were less likely than men to be honored with endowed chairs—and women who were honored with endowed chairs had higher citation indices (among other performance metrics) than men with endowed chairs (Treviño et al., 2018). Professional visibility is also a problem for scholars of color. In a study of psychology journals from 1974 to 2018, 93% of editors in chief were White (including 100% in cognitive psychology; Roberts et al., 2020, p. 5). Similarly, researchers found majority White editorial boards in communication journals (Chakravarty et al., 2018).

At the same time that women and people of color are less professionally visible, there is evidence that performing at too high a level as a woman or a historically marginalized scholar can have negative ramifications. In an experimental study of manipulated job applications, a researcher discovered that high-achieving women were less likely to be called back than high-achieving men (Quadlin, 2018). A survey of potential employers revealed a preference for likeable women over smart women (Quadlin, 2018). In another study, more expert women were seen as less-expert, and were less likely to be listened to, than less-expert women on a group task; in contrast, there was no difference in expertise perception for men, and more-expert men were listened to more than less-expert men (Thomas-Hunt and Phillips, 2004). This meant that teams with expert women were unable to capitalize on the team’s expertise.

Recognition decisions are made on numerous criteria. How to weigh the different criteria is an inherently subjective process. In addition, professional recognition is often based on whose name springs to mind when a small committee is thinking about whom to invite to serve on an editorial board or whom to honor with an award. Because there is no reason to believe that women or people of color are less deserving of recognition, extra effort is needed to identify potential candidates and to ensure that their contributions are properly weighed.

#### Disparity in Funding

Disparate funding is another concern. An analysis of publicly available NSERC funding data in Canada showed that women in cognitive psychology and cognitive neuroscience received smaller investigator-initiated Discovery grants than their male counterparts (Titone et al., 2018). In the United States, funding allocations have been argued to be about research topic choices. For example, because community-level studies are funded less by NIH than studies about the mechanism behind an effect, Black scholars who prefer community-level studies get fewer grants (Hoppe et al., 2019). But researchers who took a close look at NIH R01 applications from 2014 to 2016 using a matched-sample design (e.g., matching on gender, ethnicity, and career stage, among other variables) showed that reviewers gave Black scholars lower scores than White scholars (Erosheva et al., 2020), a result also observed in a study of NIH R01 applications submitted between 2011 and 2015 (Hoppe et al., 2019). Black scholars were also less likely to be awarded R01s between 2000 and 2006 (Ginther et al., 2011). Once again, the discrepancy in funding is not necessarily a result of overt bias, but it does signal a need for making reviewers more aware of how biases can affect their decision making.

#### Disparity in Recommendation Letters

The way research is assessed and valued is a big part of the problem in promoting the scholarship of historically marginalized faculty. As people tasked with assessment, recommendation letter writers are key to an applicant’s success. But this process is also prone to bias. In one study, researchers did a content analysis of recommendation letters written for medical school faculty in the mid-1990s, finding that recommendations for women included more language that raised doubts (Trix and Psenka, 2003). Other researchers who controlled for the productivity and postdoctoral experience of applicants also found that recommendation letters for women to assistant professor jobs had more doubt raisers than letters for men. They further found that doubt raisers decreased evaluations of competence—even when the doubt raiser was only one sentence in an otherwise positive letter (Madera et al., 2019). Doubt raisers were sentences like, “She is unlikely to become a superstar, but she is very solid” and “I assume she will be a relatively good teacher of undergraduate and graduate students” (Madera et al., 2019, p. 294). The doubt raising happens not only in the recommendation letters, but also in review of files. In an experimental study of how 238 psychologists reviewed curriculum vitae, researchers found that the psychologists made four times more doubt-raising comments about a CV with a female name compared to an identical CV with a male name (Steinpreis et al., 1999). There were comments like “we would have to see her job talk” and “I would need to see evidence that she had gotten these grants and publications on her own” (Steinpreis et al., 1999, p. 523). The experimental study points to bias as the cause of the discrepancy observed in content analyses of recommendation letters.

Other aspects of language use also vary systematically across recommendation letters written for female and male applicants. Recommendation letters for female medical school faculty highlighted teaching rather than research (Trix and Psenka, 2003). For example, two of the most common terms associated with the pronoun *her* were *training* and *teaching*, but of the pronoun *his* were *research* and *skills and abilities* (p. 211). Other researchers found that male applicants to psychology faculty positions were more likely to be described with active and assertive words (*confident*, *independent*, *outspoken*) rather than social or emotive words (*helpful*, *nurturing*, *caring*)—and the more active and assertive the descriptors, the higher the applicant was evaluated (Madera et al., 2009, p. 1593). Still others analyzed over a thousand postdoctoral recommendation letters from 54 countries collected from 2007 to 2012, finding that men were twice as likely to get excellent as opposed to good letters compared to women (Dutt et al., 2016).

Like professional recognition, recommendation letters are inherently subjective. Because there is no reason to believe that women as a group perform worse than men, we conclude that extra effort is needed to ensure parity in how women and men are evaluated. We could not find literature on recommendation letters for people of color. If such work reveals disparities, this information could further help letter-writers craft more equitable recommendation letters.

#### Disparity in Evaluation of Training Institutions

Recommendation letters are largely beyond a scholar’s control. Another aspect of research quality assessment that is beyond a scholar’s control is the prestige of the institutions they are affiliated with. The prestige of the institution where a faculty member trained has an inordinate pull on their future careers. A quarter of the institutions produced over 70% of the tenure-track faculty, and, at most, 14% of faculty get jobs at institutions that are more prestigious than where they earned their PhDs (Clauset et al., 2015). There is a gender component to many of these placements. The researchers looked at 19,000 faculty in three disciplines. They found that in computer science and business, men land in more prestigious places; in history, which has more female scholars, this pattern was not observed (Clauset et al., 2015).

But the quality of a scholar’s work is more influenced by where they land than where they trained (Way et al., 2019). Researchers looked at productivity metrics from the 5 years pre-hire and 5 years post-hire of over 2,400 early career scholars at 205 PhD-granting computer science departments in the United States and Canada from 1970 to 2011. They used a matched-samples design to compare scholars from lower-ranking and higher-ranking institutions. While being trained at a prestigious institution did lead to more citations, it did not lead to greater productivity (Way et al., 2019). At the same time, people who landed at more prestigious institutions produced an average one more paper per year, five more over 5 years, and garnered more citations (Way et al., 2019). The authors rejected three alternative explanations for their observations: that scholars hired at prestigious institutions were selected because they were more productive, that scholars at prestigious institutions adapt their productivity to match their peers at the new institution, and that prestigious institutions are more likely to retain productive faculty (or let go of unproductive faculty). Instead, they argued that prestigious universities have more research support, such as more doctoral students per faculty member, or optimal department sizes to spread the service load and allow time for research (Way et al., 2019).

#### Other Disparities

There are many other marginalized identities that can affect research assessments of women and scholars of color in an intersectional way. One example of a factor that affects women differently from men is weight. In a study of 97 prospective graduate students, those with a higher body mass index were less likely to receive an offer of admission after an in-person interview compared to prospectives who were interviewed by phone (Burmeister et al., 2013). While weight bias affected everyone, it affected female applicants more (Burmeister et al., 2013). White female college students with higher body weight reported that their families contributed less money for their education than those with lower body weight; this difference was not observed in White male college students (Crandall, 1995). In a study using actors who wore prosthetics to make them appear heavier, the heavy female job candidate was less likely to be offered a job than the heavy male candidate (Pingitore et al., 1994). In an Italian study using CVs and photos, researchers also observed that weight bias was worse for women (Busetta et al., 2020).

Still other marginalized identities that could intersect with gender and race include class, religion, able-bodiedness, sexual orientation, gender identity, immigration status, language factors such as accents, and whether or not the scholar is a parent. More research is needed to examine the interplay of these important variables.

#### A Note on Impact Metrics

Before leaving the topic of differential assessment of research, we would like to highlight a factor that is absent in most discussions of the quality of an academic candidate’s research: the societal impact of a scholar’s work. Societal impact is often a big part of the work of historically marginalized scholars, but societal impact is not always recognized by others. This point was powerfully made by the observation that Mamie and Kenneth Clark, whose research on Black children’s responses to black and white dolls was integral to the Brown versus Board of Education ruling, were not included in a compendium of prominent psychologists (Zárate et al., 2017).

### Teaching Assessment

Another significant challenge in increasing representation of women of color in the professoriate arises from evaluations of teaching quality. Student evaluations of teaching play a big role in hampering careers, as illustrated by this comment to a *Chronicle of Higher Education* article on Black women and tenure: “If the subjective opinions of 18 year-olds continue to weigh in on our career paths, then tenure will remain not only elusive, but destabilizing” (Chambers, 2011, p. 244). Disparities in student-teacher interactions further burden historically marginalized faculty. Bias affects many aspects of teaching quality assessment.

#### Disparity in Student Evaluations of Teaching

The primary tool for teaching assessment is student evaluations of teaching (SETs). SETs are important because they give all students in a class an opportunity to flag important concerns that might otherwise not be noted, such as whether an instructor has used biased materials in their lectures. At the same time, student evaluations of teaching are known to reflect gender and racial biases of the evaluators (for a review, see Heffernan, 2021). In one convincing study, an on-line class was taught by a male and female instructor with either male or female on-line identities; the students rated both the actual and perceived female instructors lower (MacNell et al., 2015). The authors provided this compelling example of the bias: “For example, when the actual male and female instructors posted grades after 2 days as a male, this was considered by students to be a 4.35 out of 5 level of promptness, but when the same two instructors posted grades at the same time as a female, it was considered to be a 3.55 out of 5 level of promptness” (p. 300). Bias is also observed for faculty of color. Across 14 on-line sections with nearly identical instruction, women and faculty of color got worse student evaluations (Chávez and Mitchell, 2020). The only interaction with students in this study was a welcome video.

Some of this bias may stem from *role incongruity*: women are expected to be nurturing, but it is hard to be nurturing on an individual level with a large lecture class, so their performance is downgraded for failure to meet expectations (Martin, 2016). In support of this, a comparison of SETs at two research universities with data from 2007 to 2014 revealed greater gender disparities with larger class sizes (Martin, 2016).

A large-scale study of almost 20,000 evaluations in the Netherlands documented that the bias against women faculty was driven by male students (Mengel et al., 2019). The bias against women instructors extends to course materials: in an online course where the materials were constant, courses taught by women had materials rated lower than courses taught by men (Mengel et al., 2019). The researchers argued that poorer SETs could lead to women faculty re-allocating their time to improving their courses, even when the evaluations are lower because of bias, not because they are worse teachers. This could result in fewer research publications for women, or more women leaving academia because of demoralization (Mengel et al., 2019).

#### Mismatch Between Evaluations and Learning

In addition to being biased, there is evidence that SETs do not even measure teaching quality. In a study comparing 23,000 French university SETs to performance on a standard exam (stratified by course subject), researchers observed no relationship between students’ ratings of learning and their actual learning in four subjects (Macroeconomics, Microeconomics, Political Science, and Sociology; Boring et al., 2016). There was a relationship in History, however (Boring et al., 2016). Despite their much higher SETs, male instructors’ students did not perform better on the exams than female instructors’ students, a finding that was also found in a United States dataset (Boring et al., 2016). Of note, male students rated male history instructors as much more effective, but actually learned more from female instructors (Boring et al., 2016).

Mismatches arise because SETs are affected by many factors besides teaching quality. One of the primary things SETs measure is the expectation of a good grade, but they also measure how science and math-oriented a course is, with worse evaluations for science and math courses (for review, see Boring et al., 2016; Heffernan, 2021). SETs are also affected by the room students take the class in; students thought instructors were more organized and coherent, and that they learned more new things, when they were in an upgraded classroom as opposed to a standard classroom (Hill and Epps, 2010).

#### Disparity in Student-Teacher Interactions

SETs are not the only problem. Students often bring their biases to the classroom, making teaching harder for historically marginalized faculty. An analysis of 17 interviews with women faculty of color at a predominantly White institution revealed that White male students had trouble accepting the faculty members as instructors with skill and wisdom, including offering advice to the faculty members about how they should do their jobs (Pittman, 2010). Other researchers found that students asked female professors to do more for them than male professors and were more upset with female professors who failed to comply with special requests (El-Alayli et al., 2018). The extra work included expected work, like asking course questions before or after class, as well as emotional labor, like discussing personal issues. Special favors included requests for personal lecture notes, requests for exceptions to course requirements, and expectations for an issue to be dealt with during an unscheduled office visit. We suspect that women faculty of color would experience even more extra workload as well as more negative responses for lack of compliance (see Carter-Sowell et al., 2019, for further discussion of this issue).

### Service Assessment

Another challenge in increasing representation of women of color in the professoriate is in evaluating service quality. Service comprises a sizeable chunk of what faculty are expected to do, yet faculty are rarely rewarded based on their service alone. Compared to readily available and widely consulted metrics that quantify research productivity and impact, efforts to quantify service contributions of faculty have proved elusive. Faculty engage in service in a variety of ways, e.g., through serving on department or university committees, on federal funding agency review panels, or on editorial boards. Service can also involve community engagement and formal or informal mentoring of students or other faculty. In recent years service has also come to include expectations for doing diversity related service. Bias affects many aspects of service quality assessment.

#### Disparity in Service Loads

Service loads are inequitably distributed across gender, making it harder for women to become promoted to full professor because they often end up, particularly after tenure, in service-heavy roles that leave little time for research (see Misra et al., 2011). In two surveys, one from 140 institutions and another from two campuses of a multi-campus institution, of over 20,000 faculty combined, women reported spending more hours per week in service activities as well as contributing to a wider range of service activities (Guarino and Borden, 2017). Differences in service can be largely attributed to women doing more internal service than men, and was described as “taking care of the academic family” (Guarino and Borden, 2017, p. 690). In another study, twenty-six faculty in five departments (including social science, natural science, and humanities) kept track of their time spent on service activities in weeks three and eight of a 10-week quarter in 2009 (Social Sciences Feminist Network Research Interest Group, 2017). In this small sample of faculty who agreed to participate in the service activity of keeping track of service activities (a point made by the authors, Social Sciences Feminist Network Research Interest Group, 2017, p. 240), assistant professor women spent more time on service than assistant professor men. But marginalized faculty (including faculty of color, queer faculty, and faculty from working class backgrounds, who made up 14 respondents) did four times the service work of White faculty.

### Shifting Criteria

A lack of consistency or transparency in the criteria used in any kind of assessment of faculty work is another source of bias that may adversely impact women of color. Shifting criteria is a problem that begins during the hiring process. In a series of experiments, researchers showed that bias creeps into hiring processes after knowledge of a candidate’s personal attributes, such as gender (Uhlmann and Cohen, 2005). The requirements for a job were redefined to fit the stereotypical applicant. For example, education was evaluated as important for applicants to the male-stereotypical job of police chief, but if the male candidate was not educated the importance of this variable was discounted; there was no difference for female applicants. In addition, the more objective evaluators thought they were, the more biased their judgements. The researchers showed that determining the importance of a criterion, such as education level, *before* evaluating candidates can eliminate bias (Uhlmann and Cohen, 2005). In practical terms, however, it can be hard to get down on paper exactly what search committees are looking for, and candidate preferences are often idiosyncratic (White-Lewis, 2020).

Shifting criteria can also affect careers in a long term sense. Interviews with academic medicine faculty revealed that criteria changed as women advanced through the ranks (Murphy et al., 2021). For example, one woman described fulfilling a requirement only to discover a new requirement; another described not being rewarded for an achievement that would have been rewarded elsewhere, receiving an R01 grant (Murphy et al., 2021).

## Possible Fixes

In the remainder of this paper we consider possible solutions to counter the biases we have identified. These are drawn from both evidence-based strategies and from our own experiences as longstanding faculty. Our list is not intended to be definitive but simply a starting point. We also acknowledge the importance of fixes that others have brought up, such as social support networks, and equitable and transparent distribution of workloads (Liu et al., 2019).

Before we get into our proposed fixes we would like to bring up an issue that is relevant here. Among senior scholars we have talked to, those who have raised issues about gender or race-related disparities in evaluation or workload have been told various versions of “this is in your head.” For example, in response to the feeling that the publication bar is set higher for them, or that students are not evaluating their teaching fairly, or that they are doing more service work than others, scholars of color have been told that what they are experiencing is not the case. They are told that instead of complaining, they should focus on improving the quantity or quality of their research, redesign their courses to please students, and continue their service activities without grumbling—or even to engage in more service activities.

Relatedly, another common response to pointing out racism and sexism is for scholars from majority groups who are just being made aware of the racism or sexism to interpret the comments about institutional practices as *ad hominem* attacks. This effectively has a silencing effect on any further discussion, particularly discussion that might have been initiated by scholars of color. Similarly, when women (particularly women of color) file formal complaints (against sexual harassment by a colleague, for example), the complaints are often trivialized and the women are seen as trouble makers (Ahmed, 2021). Thus, one thing that institution leaders could do is to regularly seek feedback from women or scholars of color in their university and not respond to it in a defensive way. Validating the lived experiences of women scholars of color is an important step in addressing equity issues. We turn next to our other possible fixes.

### Use Structured Free Recall in Assessment

One way evaluators can gird themselves against biased thinking when assessing graduate school candidates, postdoctoral candidates, or faculty candidates is by using *structured free recall* in assessment: Using the evaluation criteria as a guide, evaluators spend five minutes noting the positives of a candidate and five minutes noting the negatives (in either order), and then use these lists when discussing candidates (Baltes and Parker, 2000; Bauer and Baltes, 2002). In laboratory experiments, structured free recall reduced bias against female professors’ teaching quality (Bauer and Baltes, 2002). It has also been successfully demonstrated to reduce bias against female leaders (Anderson et al., 2015), as well as bias against Black male managers (Baltes et al., 2007). The technique has also shown promise in reducing other kinds of bias, such as bias against people who weigh more (Rudolph et al., 2012).

What structured free recall does is that it forces evaluators to recall both biased-consistent memories as well as bias-inconsistent memories, which allows for a fairer assessment (Baltes et al., 2007). Avoiding idiosyncratic feelings is important, so evaluators should work to remember specific examples of candidate’s behavior (Anderson et al., 2015). Evaluators should also recall both positive and negative information, as merely recalling details about candidates (unstructured recall) does not successfully reduce bias (Baltes et al., 2007).

Ethics precludes testing this method experimentally with actual faculty hiring; in real hiring, all candidates must be evaluated with the least biased method possible. A before-and-after field method might prove informative, but has not been carried out to date. Given the potential utility of this technique and the variety of biases evaluators can hold—with a range of strengths—we believe it is worth considering employing this technique when making hiring decisions.

### Look Beyond the Standard Metrics of Impact (But Also Be More Mindful of Whose Work is Being Erased by Not Being Cited)

Standard metrics of impact (such as journal impact factor or the scholar’s *h* index) are, ultimately, proxy indicators of impact and operationalize impact in a specific way (number of citations). They should not be taken as the final word on a scholar’s impact in a field. At the same time, given the importance that citation impact is typically given in promotion decisions, professional societies should establish formal guidelines or rubrics to promote equity in who gets cited. Further, journal editors and reviewers should hold authors accountable for their citation choices. Placing more emphasis on the originality or creativity of the work instead of on its number of citations is another way to level the playing field in evaluating a researcher’s record (cf. Hofstra et al., 2020). Other informative indicators could be to ask candidates for faculty positions about how they see their work addressing broader issues in the field (Bhalla, 2019). Encouraging a broader scope of research approaches and topics could be explicitly noted in the job ad. In addition, valuing a scholar’s impact on public policy or presence as a public intellectual could also help redress the undervaluing of minoritized scholars’ work. One alternative metric that has been looked at was how often people read articles using Mendeley bibliographic software (Thelwall, 2018). Looking across 82 fields in 2014, Thelwall found that female first-authored papers were more read than male first-authored papers in the United States. Other metrics like Altmetric and PlumX may also help properly reward research that has a public impact.

### Ensure a Broad Applicant Pool and Frame Job Ads More Inclusively

Ensuring the applicant pool is more broadly representative may also improve hiring decisions. When MBA students judged applicants for a managerial position, if the applicant pool had 12.5% or 25% female applicants (1 or 2 in a set of 8), the applicants were evaluated as less suitable and were thought of in more gender-stereotypical ways than when the female applicant represented 37.5, 50, or 100% of the pool (Heilman, 1980). Good practice would be to review the candidate pool for an advertised position and allow a search to go forward only if there is sufficient evidence that the job has been widely advertised and has a sufficiently diverse pool of applicants. Proactively reaching out to job candidates is essential, such as reaching out to professional societies for women or people of color (Wingfield, 2020).

### Reward Mentoring and Other Forms of Service

Creating effective mentoring matches is another way to enhance equity and potentially avoid some of the pitfalls of low publication rates. There is some evidence that matching a historically marginalized graduate student with a historically marginalized faculty member improves the graduate student’s productivity: Women graduate students at Caltech in the late 2000s published more with female advisors than with male advisors (Pezzoni et al., 2016). To support the retention of women of color faculty, an effective university-wide mentoring program was instituted at Texas A&M University as part of an NSF-funded ADVANCE Center grant. In this program, women faculty were assigned an internal advocate (a senior faculty member who could help them navigate the tenure process within the university) and an external eminent scholar in their field who mentored them on how to achieve professional visibility (see Carter-Sowell et al., 2019). Relatedly, rewarding faculty on the basis of the number of students they have mentored is also important (Zárate et al., 2017).

Service should be considered seriously in hiring and promotion. For example, diversity statements could be evaluated early in the hiring process, even as the first step before other material is evaluated (Bhalla, 2019). As another example, the ability to balance research, teaching, and service could be treated as a plus (Bhalla, 2019). An idea offered by a reviewer of this paper was to compensate internal service with course buyouts.

### The Value of Workshops

Attending diversity workshops has been shown to improve equity on campuses. In two studies from 2012 to 2016 at the same university, researchers assessed how attending a 2 hour equity workshop affected endorsement of strategies that promote equity, such as deciding on evaluation criteria before beginning a search and creating a more diverse search committee (Sekaquaptewa et al., 2019). Not only did faculty who attended workshops endorse the strategies more, departments with a higher percentage of faculty who had attended the workshops endorsed the strategies more—even if the faculty members themselves did not attend a workshop. Although attitudes towards some strategies were harder to change than others [most notably those that dealt with bias more directly, such as “Avoid interviewing only one candidate from a particular social group (e.g. gender or race)”], change could be achieved with increasing endorsement of social science principles, such as “Our assumptions about a person’s traits and abilities can subconsciously influence hiring decisions” (Sekaquaptewa et al., 2019; p. 199; p. 200).

In another study, researchers used a three-step process to improve hiring of women in STEM, including strategies for broadening searches, a 30-min talk on implicit bias, and availability of a faculty mentor who also helped answer applicant questions about family policies (Smith et al., 2015). The interventions were tested by randomly assigning search committees to an intervention or no intervention group. The no intervention group received training from HR on topics such as avoiding a discrimination lawsuit. Not only were women candidates more likely to get job offers, they were also more likely to accept job offers when their search committees had been in the intervention group (Smith et al., 2015).

### Sometimes, Any Fix is Better Than No Fix

There is reason to believe that any policy change will improve things for female scholars and scholars of color. Researchers looked at how often articles in the top twenty law review journals were cited from 1960 to 2018 (Chilton et al., 2022). They discovered that the implementation of a diversity policy at the journal resulted in higher-cited articles (Chilton et al., 2022). The policies implemented were diverse, such as thinking about diversity when constructing an editorial board or considering diversity statements in selecting law review members. The fact that the policy itself did not matter suggests that at least sometimes any fix is better than no fix. In the case of the law review journals, all authors saw citations benefits, regardless of whether they were women scholars of color or not.

## General Discussion

There are many challenges to the hiring of historically marginalized faculty. Bias can creeps into all aspects of assessment—research, teaching, and service. It can also affect retention and how respected and valued scholars feel about their research, teaching, and service.

Our analysis of the challenges to attracting, advancing, and keeping women of color in academia has special relevance for our field, psychology. While gender parity in psychology is good (American Psychological Association, 2006), the percentage of women full professors in psychology is not on par with men (American Psychological Association, 2014). Furthermore, the social sciences lag other fields in racial diversity (Hur et al., 2017); psychology faculty are 78% White (American Psychological Association, 2019). Extrapolating from this data, we conclude that women of color are underrepresented in psychology, especially at higher ranks. We have described in the current report some of the challenges faced by women of color in academia. By acknowledging and addressing these challenges, we can improve women faculty of color’s representation among faculty, their equity in treatment, and the recognition of their work.

Bias in research assessment can arise from the uncritical use of proxy indicators of productivity and impact and from the lack of access to cauthorship networks or mentoring relationships that enhance a scholar's professional visibility. It is hard to escape the observation that historically marginalized faculty have to work harder to achieve the same level of professional visibility as non-marginalized faculty. One of the most disheartening findings with respect to research recognition is that despite their contributions that moved their fields along, women and people of color did not see commensurate professional rewards (Hofstra et al., 2020). We believe that rewarding innovation and considering alternative metrics of success can help faculty reap the professional rewards of their work.

Bias in teaching assessment arises both from the uncritical reliance on student teaching evaluations as well as from students challenging the legitimacy of their instructor in the classroom. Historically marginalized faculty in particular experience devaluing of their skills and knowledge from their students. Once again, it is hard to escape the observation that despite a mountain of documentation that they are biased instruments, and that they do not even measure how well students learn material, student evaluations of teaching are nonetheless routinely used in hiring and promotion decisions. Given that the hiring of women faculty and faculty of color has stayed stagnant over the last decade (e.g., Bennett et al., 2020), relying on biased teaching assessment can come across to historically marginalized faculty as a feature rather than a bug—a way to not promote women and faculty of color. Being required to read and address comments in biased evaluations can also be seen as inequitably detrimental to careers, both because of the hurtful things students write to historically marginalized faculty, but also because marginalized faculty will expend more energy fixing classes that don’t need fixing—energy that could be spent on research. Proposals to optionally include additional information alongside SETs (e.g., see Center for Innovations in Teaching and Learning, 2018) do not fix these inequalities. The faculty who will feel the need to do extra work documenting that they can in fact teach are the same faculty who are compelled to do so because of biased SETs. Like fixing classes that don’t need fixing, assembling and preparing optional materials documenting teaching quality drains faculty members’ time for research. The continued use of a biased tool gives the tool legitimacy that it does not merit. We believe that faculty will feel less demoralized by teaching if institutions recognize that SETs are biased and that students bring their biases to the classroom.

Service bias includes greater expectations for doing diversity work (Guillaume and Apodaca, 2020; Joseph and Hirshfield, 2010). In addition, women bear more responsibility for the academic family, and getting the necessary service work done for universities to run (Guarino & Borden, 2017). We believe that faculty will have more time to complete their research and work on their teaching when they have more balanced service loads or when service is given more weight in their evaluations.

Faced with the barriers we have reviewed, it is no surprise that women and people of color feel discrimination in their jobs. About two out of five Latina/o/x scholars and three out of five Black scholars have faced some form of discrimination in their academic jobs (Pew Research Center, 2018). Women of color are particularly subject to different forms of bias, as described in a large scale interview study (Williams, 2014; see also; Chambers, 2011; Drame et al., 2012; Settles et al., 2021; Zimmerman et al., 2016). Focused effort will be required to make hiring and promotion equitable. In addition to implementing corrective action to counteract the effect of bias, widespread knowledge of bias in the academic system can help in supporting historically marginalized scholars. The processes that make hiring and promotion harder for historically underrepresented scholars have been operating for generations. Yet research documenting the biases is weighted towards more recent years.

The good news is that biases can be faced head-on and efforts can be made to counteract them. Acknowledging and validating the experiences of women faculty of color is a first step. Using hiring strategies shown to mitigate bias is another step. Recognizing the research achievements of women of color is yet another step, such as through greater attention to their research work and more professional recognition. Perhaps the most difficult change is to implement corrective action for unjust practices, such as by prizing research innovation over research training locale (Hofstra et al., 2020; Way et al., 2019), removing reliance on SETs in evaluating teaching (as recommended by Boring et al., 2016), and distributing service work more equitably (Guarino and Borden, 2017).

Efforts to correct unjust practices would go a long way to addressing retention of women and faculty of color which, in turn, will benefit future generations of scholars and students. With better retention, historically marginalized scholars are less likely to be the only representative of their group in a department. People are happier when their identities are more represented in a department. In a survey of faculty in STEM departments in the same university, women were less happy with their jobs than men in departments where they made up less than 25% of the faculty; where they were closer to 50%, there was no difference in job satisfaction (Griffith and Dasgupta, 2018). Greater representation in a department may also help lessen stress due to discrimination, which is particularly noteworthy for faculty of color (Eagan and Garvey, 2015). Researchers studied about 20,000 faculty across over 400 institutions who completed the Higher Education Research Institute’s survey that included measures of faculty stress and productivity (Eagan and Garvey, 2015). Black and Native American faculty had the lowest research productivity (a third of a standard deviation below White productivity) and women also had lower research productivity compared to men (a 10th of a standard deviation below). The more stressed faculty of color felt, the more their research productivity declined. In addition to improved faculty morale with greater representation, more diverse role models are likely to attract more diverse people into academia. When a student sees a professor who looks like her, she may be more likely to consider becoming a professor herself.

The pace of institutional change is notoriously slow, and academic institutions in the United States have been especially slow to respond to the growing diversity of the student body and to its demands that the university diversify its professoriate. The resistance to change on the part of senior faculty in gatekeeping positions may reflect an uncritical adoption of standards of merit that were set in place by and for a once dominant and still highly influential segment of the professoriate. Yet it is important to recognize that merit, like many other aspects of life, is a social construction. Diversifying the professoriate will require an examination of how the rhetoric of meritocracy has been used to maintain racial and gender hierarchies and inequities.
